# Prognosis and incidence of infections in chronic kidney disease patients with membranous nephropathy enrolled in a large Japanese clinical claims database

**DOI:** 10.1186/s12882-023-03190-6

**Published:** 2023-05-05

**Authors:** Takuro Matsuzaki, Yu Watanabe, Akihito Tanaka, Kazuhiro Furuhashi, Shoji Saito, Shoichi Maruyama

**Affiliations:** 1 Department of Nephrology, Nagoya University Graduate School of Medicine, Nagoya, Aichi Japan; 2 Department of Nephrology, Nagoya University Hospital, Nagoya, Aichi Japan

**Keywords:** Membranous nephropathy, Nephrotic syndrome, Steroids, Immunosuppressive agents, Infection

## Abstract

**Background:**

The treatment of membranous nephropathy involves a combination of conservative approaches, steroids, and immunosuppressive agents. Infection is an adverse effect of these treatments and its incidence is a critical issue for patients with membranous nephropathy, as many of them are older adults. However, the incidence of infections remains unclear; hence, this study investigated this issue using data from a large Japanese clinical claims database.

**Methods:**

From a database of patients with chronic kidney disease (*n* = 924,238), those diagnosed with membranous nephropathy from April 2008 to August 2021 with a history of one or more prescriptions and undergoing medical care were included. Patients who had undergone kidney replacement therapy were excluded. Patients were divided into three groups based on their prescriptions after diagnosis: prednisolone(PSL), who received steroids; PSL + IS, who were prescribed steroids and immunosuppressive agents; and C, who were treated without steroid or immunosuppressive agent use. The primary outcome was death or the initiation of kidney replacement therapy. The secondary outcome was death or hospitalization due to infection. Infectious diseases such as sepsis, pneumonia, urinary tract infections, cellulitis, cytomegalovirus infection, colitis, or hepatitis were defined as infections. Hazard ratios were expressed using group C as a reference.

**Results:**

Of 1,642 patients, the incidence of the primary outcome occurred in 62/460 individuals in the PSL group, 81/635 individuals in the PSL + IS group, and 47/547 individuals in the C group. The Kaplan–Meier survival curve showed no significant differences (*P* = 0.088). The incidence of secondary outcomes occurred in 80/460 individuals, 102/635 individuals, and 37/547 individuals in the PSL, PSL + IS, and C groups, respectively. The incidence of secondary outcomes was significantly higher in the PSL group (hazard ratio [HR] 2.43 [95% confidence interval [CI] 1.64–3.62, *P* < 0.01]) and PSL + IS group (HR 2.23 [95% CI 1.51–3.30, *P* < 0.01]).

**Conclusions:**

The outcome of membranous nephropathy was not completely satisfactory. Patients who use steroids and immunosuppressive agents have a high incidence of infection and may require close monitoring during the course of treatment.High-efficacy treatment with a low incidence of infections is desirable. The significance of this study lies in the fact that the impressions of membranous nephropathy, which have been recognized as tacit knowledge, were quantified using a clinical database.

**Supplementary Information:**

The online version contains supplementary material available at 10.1186/s12882-023-03190-6.

## Background

Membranous nephropathy is a glomerulopathy in which immune complexes are deposited on the kidney glomerular basement membrane [1]. It is said to be the most frequent primary disease, causing nephrotic syndrome in middle-aged and older adults [2]. The prognosis of membranous nephropathy is reported to be better in Japan than in Europe and the United States. Approximately 80% of patients achieve complete remission with little progression to kidney failure [3]. Nevertheless, the long-term prognosis is not always favorable [4], yet this finding needs to be investigated as there are no reports of long-term follow-up for patients in large-scale databases.

Since the induction of remission makes a significant difference in prognosis, a treatment that can induce remission promptly and efficiently is desired. In addition to steroids, immunosuppressive agents such as cyclosporine, mizoribine, and cyclophosphamide are effective in the treatment of membranous nephropathy. According to the Japanese guidelines for nephrotic syndrome, treatment options include conservative treatment, steroid monotherapy, and combination therapy with steroids and immunosuppressive agents [5] (Supplementary Fig. 1). Immunosuppressive agents refer to those other than steroids. The guidelines state that the above three treatment strategies should be used according to the individual condition of the patient; however, there are no clear criteria for their use. Although there are deviations from the global standard of treatment, steroid monotherapy is an option included in the Japanese guidelines and has been previously reported [3, 6]. Since many patients with membranous nephropathy are older adults and spontaneous remission is common, conservative therapies such as angiotensin-converting enzyme inhibitors (ACEi) and angiotensin II type 1 receptor blockers (ARB) are often used as the treatment of choice. More importantly, the development of concomitant infection during treatment is considered a more pressing concern, as many patients with membranous nephropathy are older adults. In Japan, a report showed that kidney survival rates were 95.8% at 5 years, 90.3% at 10 years, 81.1% at 15 years, and 60.5% at 20 years [4]. Therefore, the kidney survival rate at 20 years may be considered poor.

Infection is an adverse effect of steroids and immunosuppressive agents. Furthermore, the incidence of infection is a critical issue for patients with membranous nephropathy because many of them are older adults. However, no large-scale database studies exist on the incidence of infections during the course of treatment for membranous nephropathy. It is unknown whether there is a difference in the incidence of infection when comparing steroid therapy alone, combined steroid and immunosuppressive agents, or conservative treatment without steroids or immunosuppressive agents, in accordance with the recommended treatment regimens stated in the Japanese guidelines.

## Methods

This study was conducted with ethical considerations in accordance with the Declaration of Helsinki. This study was approved by the Ethics Committee of the Institutional Review Board of Nagoya University Hospital (approval number; 2021–0350). Since no personally identifiable information was obtained in this study, the need for informed consent was waived by the approval of the Ethics Committee of the Institutional Review Board of Nagoya University Hospital. This study investigated the incidence of infections associated with the treatment of membranous nephropathy. In this retrospective cohort study, patients with membranous nephropathy, selected from a large-scale claims database, were divided into three groups: steroids alone, steroids with immunosuppressive agents, and conservative treatment without steroids or immunosuppressive agents. Kidney survival and infection rates in the three groups were investigated and compared.

### Data collection

**Fig. 1:**
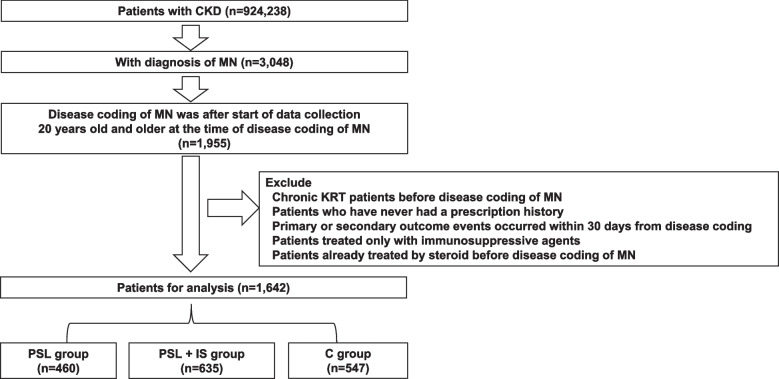
The figure shows the flowchart of patient registration. CKD, chronic kidney disease; MN, membranous nephropathy; KRT, kidney replacement therapy; PSL, prednisolone; IS, immunosuppressive agent; C, conservative treatment group

A Japaneses medical claim database on procedures, prescriptions, surgeries, hospitalizations, and laboratory data for the period from April 2008 to August 2021 was obtained from the Medical Data Vision Co., Ltd. (MDV). This data collection started from April 2008 by MDV. As of August 2021, MDV had collected 36,690,000 patient-records from 449 hospitals in Japan. From this database, we extracted patients with registered chronic kidney disease (CKD) codes. We obtained data for 924,238 patients with CKD. Among these, we selected patients with disease codes for membranous nephropathy. Diagnosis was determined by the presence of a confirmed disease code. A flowchart of patients registration can be seen in Fig. 1. Patients were divided into three groups: steroid monotherapy (prednisolone (PSL) group), steroid and immunosuppressive agent combination therapy (PSL + IS group), and conservative treatment without PSL or immunosuppressive agents (group C). Patients taking oral steroids were defined as those who were prescribed oral prednisolone. Patients taking immunosuppressive agents, including cyclophosphamide, mycophenolate mofetil, mizoribine, cyclosporine, tacrolimus, and rituximab, were included in the immunosuppressives group. Those who were not prescribed any of these aforementioned drugs were considered to have undergone conservative treatments. Patients who had been prescribed steroids before the diagnosis of membranous nephropathy were excluded in order to exclude secondary membranous nephropathy associated with other diseases. Patients prescribed dialysis without being billed for the additional induction phase of dialysis were also excluded.

### Definition of outcomes

The outcomes of this study were mortality, kidney events, and the incidence of infection. Mortality was assessed by the discharge summary, and if death was checked, the patient was considered dead. Patients who were alive and still attending outpatient clinics were considered alive as long as they continued to attend outpatient clinics. Follow up was continued as long as they continued to attend outpatient clinics. Follow up was censored if they stopped attending outpatient clinics. A kidney event was defined as the start of kidney replacement therapy, such as kidney transplantation, or the induction of hemodialysis or peritoneal dialysis. Infection was defined as sepsis, urinary tract infection, pneumonia, cellulitis, cytomegalovirus infection, colitis, or hepatitis according to the medical code at admission. For colitis and hepatitis, we include only those whose cause is determined to be infectious from the disease codes.Infection outcomes were defined as hospitalized infections only. Infections without hospitalization were not included as outcomes because they included minor infections, and it is unclear whether they are clinically meaningful. For the aforementioned infection outcomes, only those diagnosed after the diagnosis of membranous nephropathy were considered.

The primary outcome was defined as death or the initiation of kidney replacement therapy. The secondary outcome was defined as death or hospitalization due to infection. After the diagnosis of membranous nephropathy, patients who had not experienced any of the above events during a 30-day observation period were included in the analysis. Since disease codes are assigned on a monthly basis, these patients were analyzed to exclude patients whose events occurred prior to diagnosis. Disease and procedure codes are listed in Supplementary Table 1.

### Statistical analysis

Baseline patient characteristics are descriptively presented. Numerical data are presented as the mean and standard deviation. Categorical data are presented as percentages. Kaplan–Meier curves were drawn for comparisons between groups, and log-rank tests were performed. The secondary outcome, infectious disease hospitalization, and death were assessed using the Cox hazards model. Hazard ratios were presented graphically using forest plots with reference to group C. Hazard ratios were adjusted by clinically important factors, such as age, sex, history of diabetes, hypertension, cerebral infarction, myocardial infarction, chronic obstructive pulmonary disease, and admission of heart failure. The amounts of steroids used over time until outcomes were also drawn with violin plots. Steroid dose was shown as the average daily dose, calculated every 3 months. Steroids except prednisolone were converted to prednisolone. Statistical significance was set at *P* < 0.05. All analyses were performed using R software [7].

## Results

### Baseline characteristics of patients

Patient backgrounds are shown in Table 1. A total of 1642 patients (1016 men and 626 women) were diagnosed with membranous nephropathy. There were 547 patients in the conservative treatment group (group C), 460 in the steroid monotherapy group (PSL group), and 635 in the combined steroid and immunosuppressive agents treatment group (PSL + IS group). In addition, 45 patients were only prescribed immunosuppressive agents; however, they were excluded from further analysis because their treatment deviated from the Japanese guidelines, they were presumed to have special circumstances, and their numbers were smaller than those of the other groups. The mean age of each group ranged from 68 to 70 years with significant differences between the groups, and many were older adults. Hypertension was a common comorbidity in all groups. A history of diabetes was least common in the PSL group. ACEi or ARB was administered to more than 70% of the patients in each group. Since pneumonia was selected as an infectious outcome, a history of chronic obstructive pulmonary disease was also considered as an underlying pulmonary disease; however, no significant difference for this was found in each group.

**Table 1:** Baseline characteristics of the participants

Characteristic	PSL, *N* = 460	PSL + IS, *N* = 635	C, *N* = 547	*p*-value
Age (y), mean (± SD)	70 (13)	68 (11)	69 (13)	< 0.001
Male, n (%)	279 (61%)	397 (63%)	340 (62%)	0.810
Past History				
HT, n (%)	378 (82%)	540 (85%)	443 (81%)	0.160
DM, n (%)	223 (48%)	371 (58%)	229 (42%)	< 0.001
AF or AFL, n (%)	37 (8.0%)	41 (6.5%)	27 (4.9%)	0.130
COPD, n (%)	15 (3.3%)	15 (2.4%)	15 (2.7%)	0.670
Cerebrovascular disease, n (%)	3 (0.7%)	9 (1.4%)	12 (2.2%)	0.130
Cardiac event, n (%)	4 (0.9%)	8 (1.3%)	5 (0.9%)	0.860
HF, n (%)	9 (2.0%)	4 (0.6%)	10 (1.8%)	0.110
Prescription				
PSL, n (%)	441 (96%)	629 (99%)	0 (0%)	< 0.001
mPSL iv, n (%)	51 (11%)	91 (14%)	0 (0%)	< 0.001
mPSL po, n (%)	6 (1.3%)	11 (1.7%)	0 (0%)	0.002
DEX, n (%)	19 (4.1%)	3 (0.5%)	0 (0%)	< 0.001
BMS, n (%)	3 (0.7%)	2 (0.3%)	0 (0%)	0.190
MMF, n (%)	0 (0%)	11 (1.7%)	0 (0%)	< 0.001
Tac, n (%)	0 (0%)	27 (4.3%)	0 (0%)	< 0.001
CY po, n (%)	0 (0%)	29 (4.6%)	0 (0%)	< 0.001
CY iv, n (%)	0 (0%)	9 (1.4%)	0 (0%)	< 0.001
CsA, n (%)	0 (0%)	480 (76%)	0 (0%)	< 0.001
RTX, n (%)	0 (0%)	29 (4.6%)	0 (0%)	< 0.001
MZR, n (%)	0 (0%)	212 (33%)	0 (0%)	< 0.001
ACEi, n (%)	34 (7.4%)	72 (11%)	60 (11%)	0.073
ARB, n (%)	330 (72%)	477 (75%)	358 (65%)	0.001
Prescription of nutritional guidance	93 (20%)	152 (24%)	102 (19%)	0.072
Primary outcome				0.003
Initiation of KRT, n (%)	21 (4.6%)	36 (5.7%)	30 (5.5%)	
Death, n (%)	41 (8.9%)	45 (7.1%)	17 (3.1%)	
Secondary outcome				< 0.001
Admission for pneumonia, n (%)	33 (7.2%)	45 (7.1%)	13 (2.4%)	
Admission for sepsis, n (%)	7 (1.5%)	1 (0.2%)	1 (0.2%)	
Admission for UTI, n (%)	6 (1.3%)	10 (1.6%)	4 (0.7%)	
Admission for cellulitis, n (%)	0 (0%)	1 (0.2%)	0 (0%)	
Admission for CMV infection, n (%)	0 (0%)	2 (0.3%)	0 (0%)	
Admission for colitis, n (%)	3 (0.7%)	7 (1.1%)	3 (0.5%)	
Admission for hepatitis, n (%)	1 (0.2%)	2 (0.3%)	0 (0%)	
Death, n (%)	30 (6.5%)	34 (5.4%)	16 (2.9%)	
Days from diagnosis to initiation of glucocorticoids, mean (± SD)	100 (334)	45 (182)	0 (0)	< 0.001

*PSL* prednisolone, *IS* immunosuppressive agent, *C* conservative therapy, *HT* hypertension, *DM* diabetes mellitus, *AF* atrial fibrillation, *AFL* atrial flutter, *COPD* chronic obstructive pulmonary disease, *HF* heart failure, *mPSL* methylprednisolone, *iv* intravenous administration, *po* per os, *DEX* dexamethasone, *BMS* betamethasone, *MMF* mycophenolate mofetil, *Tac* tacrolimus, *CY* cyclophosphamide, *CsA* ciclosporin, *RTX* rituximab, *MZR* mizoribine, *ACEi* angiotensin-converting enzyme inhibitor, *ARB* angiotensin II type 1 receptor blocker, *KRT* kidney replacement therapy, *UTI* urinary tract infection, *CMV* cytomegalovirus

### Primary outcome: death and induction of kidney replacement therapy

**Fig. 2:**
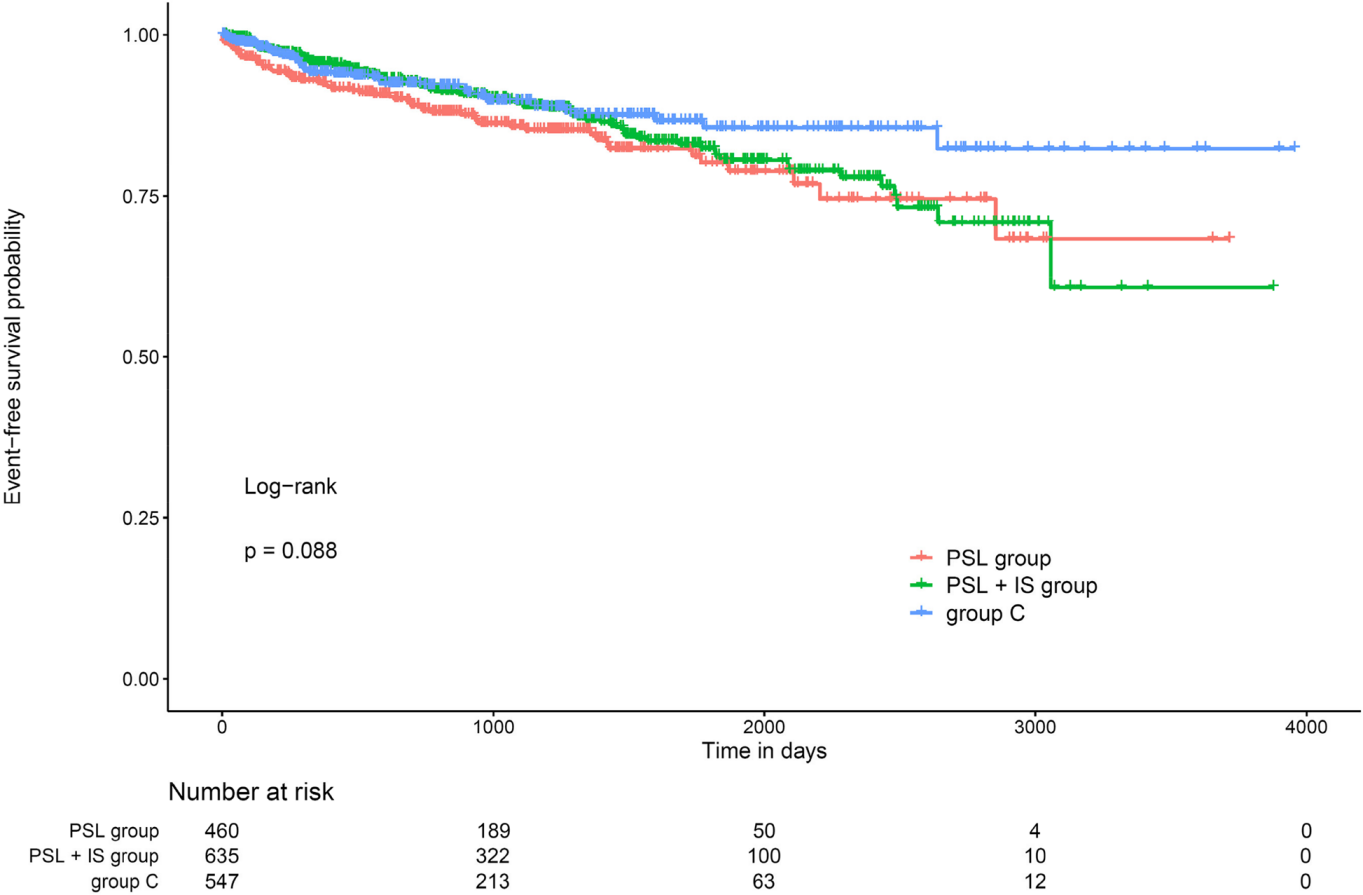
Kaplan–Meier plot of event-free survival rates in patients with membranous nephropathy. PSL, prednisolone; IS, immunosuppressive agent; group C, conservative treatment group

Figure 2 shows the event-free survival rates for the induction of kidney replacement therapy and mortality as the primary outcomes in all three groups. Although there was a trend toward higher survival in group C than in the PSL and PSL + IS groups, no significant difference was observed. Overall, the event-free survival rate was less than 80% during observation period, which was not as satisfactory as expected. Because the data on severity of illness, such as the amount of urinary protein, were not available, we did not perform multivariate analysis.

### Secondary outcome: death and admission for infectious disease

**Fig. 3:**
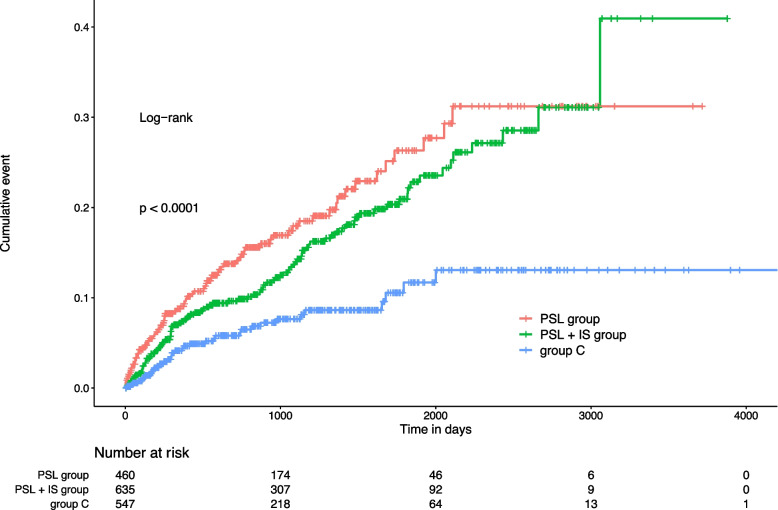
Kaplan–Meier plot of death and infection in patients with membranous nephropathy. PSL, prednisolone; IS, immunosuppressive agent; group C, conservative treatment group

The incidence of death, sepsis, pneumonia, and urinary tract infection, cellulitis, cytomegalovirus infection, colitis, or hepatitis in the three groups is shown in Fig. 3. The incidence of infection was significantly higher in the PSL (17.4%) and PSL + IS (16.1%) groups than in group C (6.8%; *P* < 0.01).

### Analysis of the association between infection and treatment intensity

**Fig. 4:**
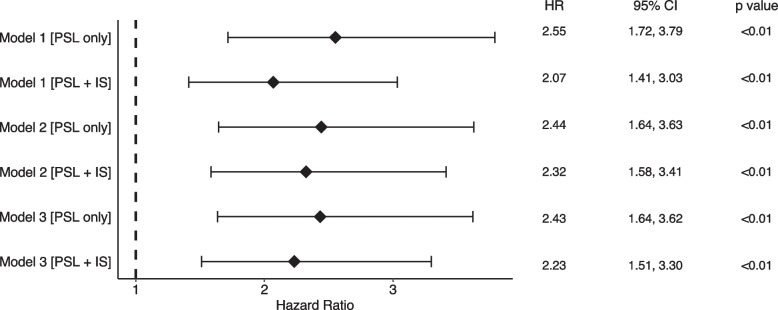
Forest plot showing hazard ratios for the secondary outcomes for the PSL and IS treatment groups. Forest plot shows the hazard ratios for secondary outcome (infections and death) were presented with reference to the C group. Model 1 was not adjusted. Model 2 was adjusted by age and sex. Model 3 was adjusted by age, sex, history of diabetes, hypertension, cerebral infarction, myocardial infarction, chronic obstructive pulmonary disease, and admission of heart failure. PSL, prednisolone; IS, immunosuppressive agent; HR, hazard ratio; CI, confidence interval

As an adverse effect, infections are a particular concern in immunosuppressive therapy. We examined treatment intensity as a factor associated with the development of infections and death. Hazard ratios were calculated for the PSL and PSL + IS groups, adjusting for various factors, and compared with group C as the reference; the results are shown in Fig. 4. Compared with group C, the PSL and PSL + IS groups had a significantly increased incidence of infection, even after adjustment for various factors (PSL group; HR 2.43, 95% CI 1.64–3.62, *P* < 0.01. PSL + IS group; HR 2.23, 95% CI 1.51–3.30, *P* < 0.01, respectively).

### Analysis of the dose of steroid use

Trends in patient steroid use were also considered an important factor and were evaluated. First, the PSL and PSL + IS groups were divided by the presence or absence of the primary outcome, respectively, and the amounts of PSL were drawn. The results are shown in Supplementary Fig. 2. First, the PSL + IS group did not have a reduction in steroid dosage, and the intensity of treatment was presumed to be stronger with the addition of IS than that of the PSL group. Therefore, the baseline severity of membranous nephropathy was likely to be different at the start of treatment. For example, at the start of treatment, the dose of PSL was 21.7 ± 17.4 mg/day in the PSL group and 24.0 ± 16.4 mg/day in the PSL + IS group. At 12 months after the start of treatment, the dose of PSL was 4.38 ± 4.05 mg/day the PSL group (*n* = 329) and 6.33 ± 5.77 mg/day in the PSL + IS group (*n* = 502). The PSL dose tended to be higher in the PSL + IS group, again suggesting a higher severity of disease. In both the PSL and PSL + IS groups, the worse prognosis group (with kidney replacement therapy or death) had a higher steroid dose at some time points, suggesting that the disease in this group was more severe. However, there was no significant difference between the groups with and without the primary or secondary outcomes. For example, at the start of treatment, the PSL group with primary outcome was 19.0 ± 17.7 mg/day and that without outcome was 22.0 ± 17.4 mg/day (*P* = 0.120). Further, the PSL and PSL + IS groups were divided by the presence or absence of the secondary outcome, respectively, and the equivalent PSL was calculated. The results are shown in Supplementary Fig. 3. Similar to the primary outcome results, the PSL + IS group did not have a reduction in steroid dosage, and the intensity of treatment was presumed to be stronger with the addition of IS than that of the PSL group. Therefore, the baseline severity of disease was still different at the start of treatment. At the start of treatment, the PSL dose was 21.0 ± 17.7 mg/day in the PSL group with secondary outcome and 23.7 ± 13.3 mg/day in the PSL + IS group with secondary outcome. Both the PSL and PSL + IS groups with the outcomes of infection hospitalization and death had a higher dose of steroids at some time points. However, there was no significant difference between the groups with and without outcomes.

## Discussion

In this study, patients with membranous nephropathy were divided into three groups: steroids alone, steroids and immunosuppressive agents, and conservative treatment. The treatment efficacy for all three groups was evaluated in terms of mortality and induction of kidney replacement therapy as a primary outcome. The efficacy of each group was not significantly different from each other, and the kidney survival rate was only approximately 75% after almost 10 years, which was not completely satisfactory. In contrast, the incidence of infection was evaluated as a secondary outcome. The incidence of infections was higher in the PSL and PSL + IS groups. The fact that the incidence of infection was lower in the conservative treatment group than in the other two groups is expected because the conservatively-treated patients were not immunosuppressed with steroids or immunosuppressive agents.

Considering data from other countries, 20%–40% of patients with membranous nephropathy develop end-stage kidney disease after 10–15 years [1, 8, 9]. Conversely, membranous nephropathy in the Japanese population is said to have a more benign course and often resolves spontaneously compared to that in the Caucasian population [10, 11]. However, in the present study, we were unable to conclude that the prognosis was better in Japanese patients than in patients from other countries.

Autoantibodies against the M-type phospholipase A2 receptor (PLA2R), which is expressed in podocytes, are causative antigens of idiopathic membranous nephropathy [12]. Anti-PLA2R antibody titers have been reported to reflect disease activity and are thought to be predictive of spontaneous remission, relapse, and decline in kidney function in membranous nephropathy. Overseas, approximately 70% of patients with idiopathic membranous nephropathy are said to be antibody-positive. However, the antibody-positivity rate in Japanese patients with membranous nephropathy is reported to be approximately 50%, which is lower than the rate in other countries [13]. This suggests that the underlying etiology of membranous nephropathy may vary between Japan and other countries. In addition, international guidelines [14] recommend that PLA2R antibodies should be measured first. However, in Japan, PLA2 antibody measurement is not covered by insurance, and the measurement system has not been established. Therefore, the level of PLA2R antibodies is not included in the data of this study, which is a limitation. The initial treatment of membranous nephropathy also differs between Japan and other countries. In Japan, oral steroids alone, conservative treatment, or a combination of steroids and immunosuppressive agents are recommended as initial treatment [5]. However, in the U.S. and Europe, the initial treatment regimen consists of rituximab, calcineurin inhibitors, and combination of corticosteroid and cyclophosphamide, with no recommendations for steroid monotherapy [14]. Therefore, owing to the varied etiologies of the disease and the vastly different treatment regimens, it is difficult to apply data from one country or region directly to all countries, and it is necessary to clarify the data from each country or region.

The results obtained in this study showed no difference in treatment efficacy between the three groups. However, caution must be exercised in interpreting these results. As the Kaplan–Meier curves showed, conservative treatment paradoxically seemed to have a better prognosis, although no significant differences were observed. This may be because group C was originally a group with mild disease which was less likely to result in the primary endpoint, which was death or the initiation of kidney replacement therapy. In contrast, the group that was selected for treatment with steroids and immunosuppressive agents may have been more severely ill and required more advanced treatment. In other words, there might have been selection bias regarding disease severity. Although we believed that adjustment for the severity of the illness was necessary, it was difficult to do so for the following reasons. First, the urinalysis and blood test data were unavailable. Therefore, it was not possible to identify the severity of the disease from the data on hand consisting of urinary protein and serum albumin levels, and as a result, it was difficult to adjust for the severity of the disease. In addition, information on the kidney biopsy results was not available. Poor prognostic factors for membranous nephropathy include advanced age (> 60 years), sex (male), poor kidney function at onset, severe proteinuria, and segmental sclerosis lesions or interstitial disorders (> 20%) in kidney pathology [4]. Data on urinalysis, blood tests, and kidney pathology findings could not be extracted from the database. Although we believe that it is highly likely that the poor prognosis group was originally biased toward the PSL and PSL + IS groups, as these findings are related to the severity of the disease and may be a factor in treatment selection, we were unable to adjust for these by multivariate analysis. Because it was impossible to adjust for the severity with the data at hand, we examined steroid dose over time as a factor that might be involved in severity. We found the intensity of immunosuppression was also likely to be higher in the PSL + IS group, and the severity of the disease was also likely to be higher than that in PSL group. Unfortunately, we were unable to adjust for disease severity as it is conceivable that it plays a role in treatment selection and prognosis. On the other hand, hospitalization for infections was significantly more frequent in the PSL and PSL + IS groups. Here, we focused on the adverse effects that occurred during treatment by performing a multivariate analysis. Even after multivariate analysis and adjustment for various factors, this association was significant. Previous reports have focused only on efficacy and have not reported on adverse effects on a large scale; therefore, it was crucial to follow up on this issue. Adverse effects are not negligible and are more worrisome in Japanese older adult patients with membranous nephropathy. In this study, only limited infectious diseases were identified as adverse effects. If other infections are included, the risk of infection would be even higher.

These results indicate that the survival and kidney event-free survival rate of patients with membranous nephropathy in Japan is not completely satisfactory. Furthermore, the current treatment regimen is associated with a very high risk of adverse effects. To overcome these unmet needs, there is a demand to establish new treatments with higher efficacy and fewer adverse effects. For example, rituximab is increasingly used overseas because of its higher therapeutic efficacy and fewer adverse effects [15]. Recently, a randomized controlled trial reported fewer treatment failures with rituximab compared to cyclosporine [16]. However, another randomized controlled trial reported no difference in efficacy between rituximab and a cyclic regimen of steroids and cyclophosphamide [17], and, one report found that a combination of tacrolimus and rituximab was not as effective as a combination of steroids and cyclophosphamide [18]. Various attempts are being made to overcome the inadequate kidney prognosis of patients with membranous nephropathy. This study is significant in that it clarifies these current conditions with unmet needs.

### Study limitations

This was a retrospective study, and the criteria for therapeutic interventions were not clear. Moreover, data on proteinuria or kidney biopsy results were not available; therefore, the results could not be evaluated with adjustments for disease severity. Since this was a database study, it was impossible to determine a causal relationship. In addition, patients treated without the use of PSL or IS were considered to be in the conservative treatment group; however, we only obtained data about the percentages of use of ACEi, ARB and nutritional guidance. Hence, the robustness of the conservative treatment provided is unclear.

This study is based on patients with the disease code of CKD. Although we know that this diagnosis of CKD has a disease code, but we cannot determine the cause of CKD. Whether CKD is due to membranous nephropathy or some other cause, as well as information on current kidney function, is unknown. It was reported that patients with CKD were more susceptible to infection [19], and it is possible that the results of this study were influenced by the inclusion of patients with decreased kidney function.

## Conclusion

We investigated the prognosis and incidence of infection in patients with membranous nephropathy using a large-scale Japanese claims database. The kidneyprognosis in these patients is not completely favorable. Patients who use steroids and immunosuppressive agents have a high incidence of infection and may require close monitoring during the course of treatment.

## Supplementary Information

**Additional file 1: Supplementary Table 1.** ICD-10 code of disease. **Supplementary Figure 1.** Treatment recommendation for membranous nephropathy from the Japanese Guideline. **Supplementary Figure 2.** Steroid dosage change in prednisolone equivalents over time until the primary outcome. **Supplementary Figure 3.** Steroid dosage change in prednisolone equivalent over time until the secondary outcome. 

## Data Availability

The datasets supporting the conclusions of this article were obtained from Medical Data Vision Co., Ltd., under an agreement not to release the original data to outside parties. Requests to access the datasets should be directed to the corresponding author, furu13@med.nagoya-u.ac.jp.

## Abbreviations

ACEi: Angiotensin-converting enzyme inhibitors
ARB: Angiotensin II type 1 receptor blockers
CKD: Chronic kidney disease
PLA2R: Phospholipase A2 receptor
PSL: Prednisolone
