# Supplementary material for: Prognosis and incidence of infections in chronic kidney disease patients with membranous nephropathy enrolled in a large Japanese clinical claims database
Source: BMC Nephrol. 2023 May 5;24:126. doi: 10.1186/s12882-023-03190-6 (PMC10161415; doi:10.1186/s12882-023-03190-6)
Supplement: Supplementary file 1 — Additional file 1: Supplementary Table 1. ICD-10 code of disease. Supplementary Figure 1. Treatment recommendation for membranous nephropathy from the Japanese Guideline. Supplementary Figure 2. Steroid dosage change in prednisolone equivalents over time until the primary outcome. Supplementary Figure 3. Steroid dosage change in prednisolone equivalent over time until the secondary outcome. [file 12882_2023_3190_MOESM1_ESM.docx]

Supplementary Table 1. ICD-10 code of disease

| Disease Name | ICD10 code |
| --- | --- |
| HT | I10, I11, I12, I13, I14, I15 |
| DM | E10, E11, E12, E13, E14 |
| AF, AFL | I48 |
| COPD | J449 |
| Cerebrovascular disease | I60, I61, I62, I63, I64, I65, I66, I67, I68, I69, G45 |
| Cardiac event | I21, I22, I23, I44, I45, I46, I47, I48, I49 |
| HF | I50, I110 |
| Pneumoniae | A010, A022, A162, A162, A403, A419, A481, A482, A491, A491, A491, A500, A548, A70, B012, B052, B068, B206, B221, B250, B59, B59, C349, C349, G001, I301, J028, J110, J120, J121, J122, J123, J128, J129, J13, J14, J150, J151, J152, J152, J152, J153, J154, J155, J156, J156, J156, J156, J156, J157, J158, J159, J160, J180, J180, J181, J182, J188, J188, J189, J189, J189, J189, J189, J189, J189, J202, J678, J679, J680, J690, J690, J690, J690, J690, J690, J690, J691, J698, J700, J704, J82, J82, J82, J82, J841, J841, J841, J841, J841, J841, J841, J841, J841, J841, J841, J849, J850, J851, J958, J958, K650, M0019, M0510, M0510, M321, M321, M330, M331, M332, M351, O290, P233, P236, P236, P239, U071 |
| UTI | A022, A181, N10, N10, N10, N111, N118, N119, N119, N12, N12, N12, N209, N390, N390, N390, N390, N390, N390, N390, O862, P393, T835, T835, T835 |
| sepsis | A021, A207, A227, A241, A267, A282, A327, A394, A400, A401, A402, A403, A409, A410, A411, A412, A413, A414, A415, A418, A419, A427, A548, B007, B349, B377, D71, I301, I330, J020, J209, J950, L029, L080, M8699, O080, O753, O85, O883 |
| Colitis | A020, A044, A045, A046, A048, A049, A052, A053, A062, A079, A080, A081, A082, A083, A084, A090 |
| CMV infection | B250, B251, B252, B258, B259, B271 |
| hepatitis | B008, B150, B159, B162, B169, B171, B172, B178, B179, B181, B182, B189, B190, B251, B268, B270, B338, B581 |

HT: hypertension

DM: diabetes mellitus

AF: atrial fibrillation

AFL: atrial flutter

COPD: chronic obstructive pulmonary disease

HF: heart failure

UTI: urinary tract infection

CMV: cytomegalovirus

Supplementary Figure 1. Treatment recommendation for membranous nephropathy from the Japanese Guideline


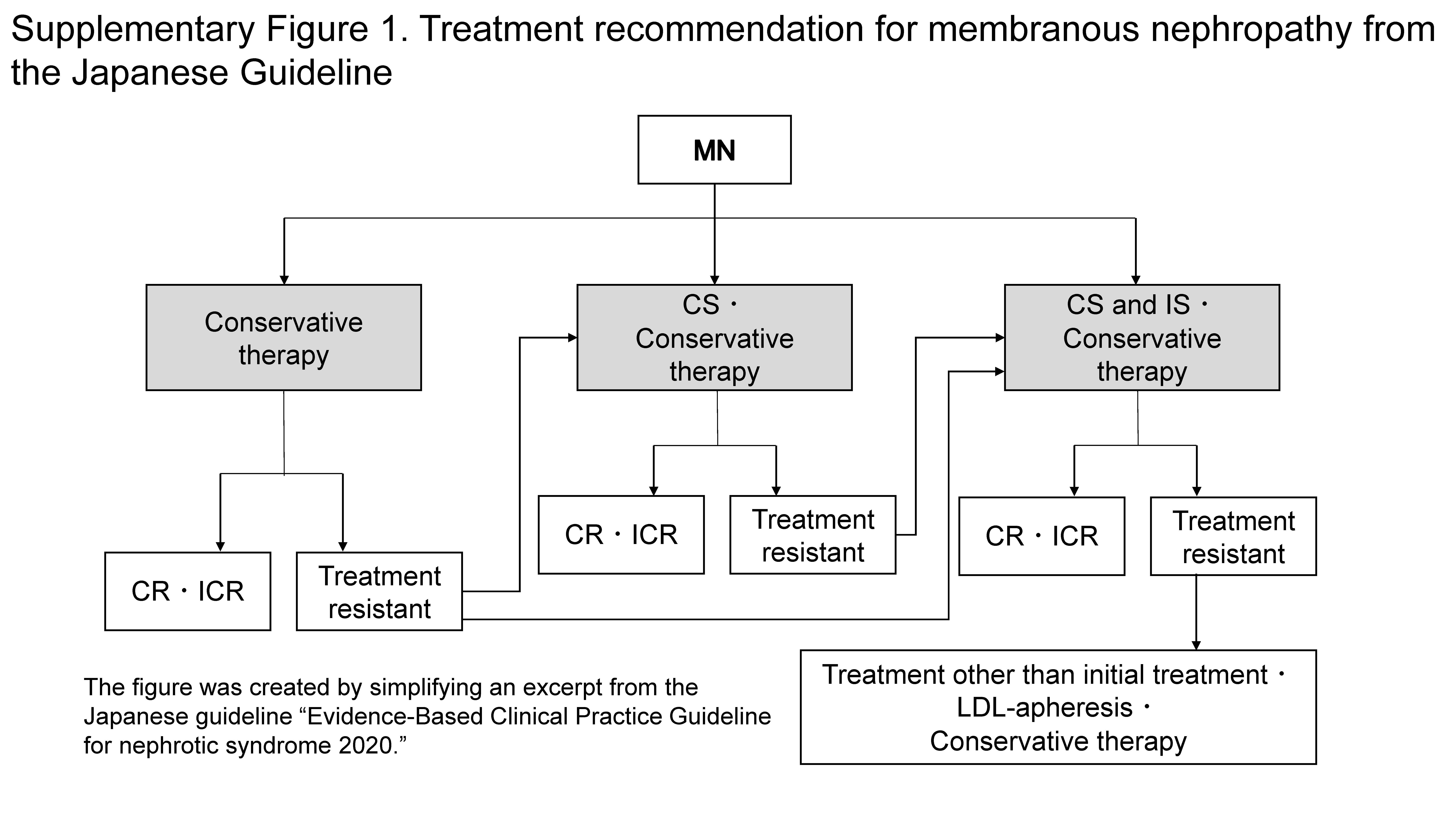


The figure was created by simplifying an excerpt from the Japanese guideline “Evidence-Based Clinical Practice Guideline for nephrotic syndrome 2020 [5].” After diagnosis, the three treatment strategies are listed in parallel, with no preference given to any of them. A treatment strategy should be selected according to the individual condition of the patient; however, there are no clear criteria for their use.

MN, membranous nephropathy; CS, corticosteroid; IS, immunosuppressive agents; CR, complete remission; ICR, incomplete remission; LDL-apheresis, low density lipoprotein apheresis.

Supplementary Figure 2. Steroid dosage change in prednisolone equivalents over time until the primary outcome


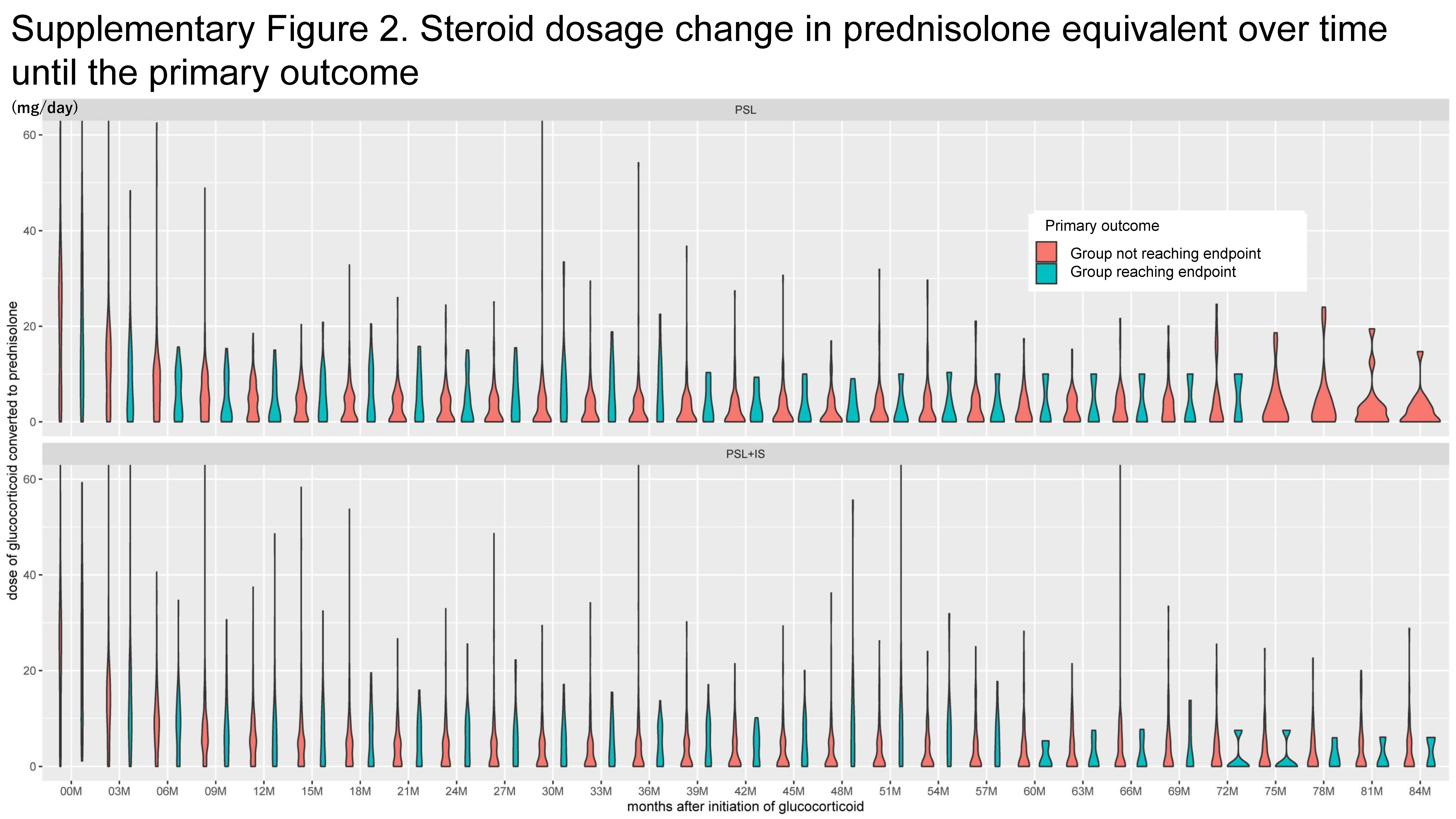


The blue area shows the group reaching the endpoint. The red area shows the group not reaching the endpoint. The upper panel shows the PSL group. The lower panel shows the PSL + IS group.

PSL, prednisolone; IS, immunosuppressive agent.

Supplementary figure 3. Steroid dosage change in prednisolone equivalent over time until the secondary outcome


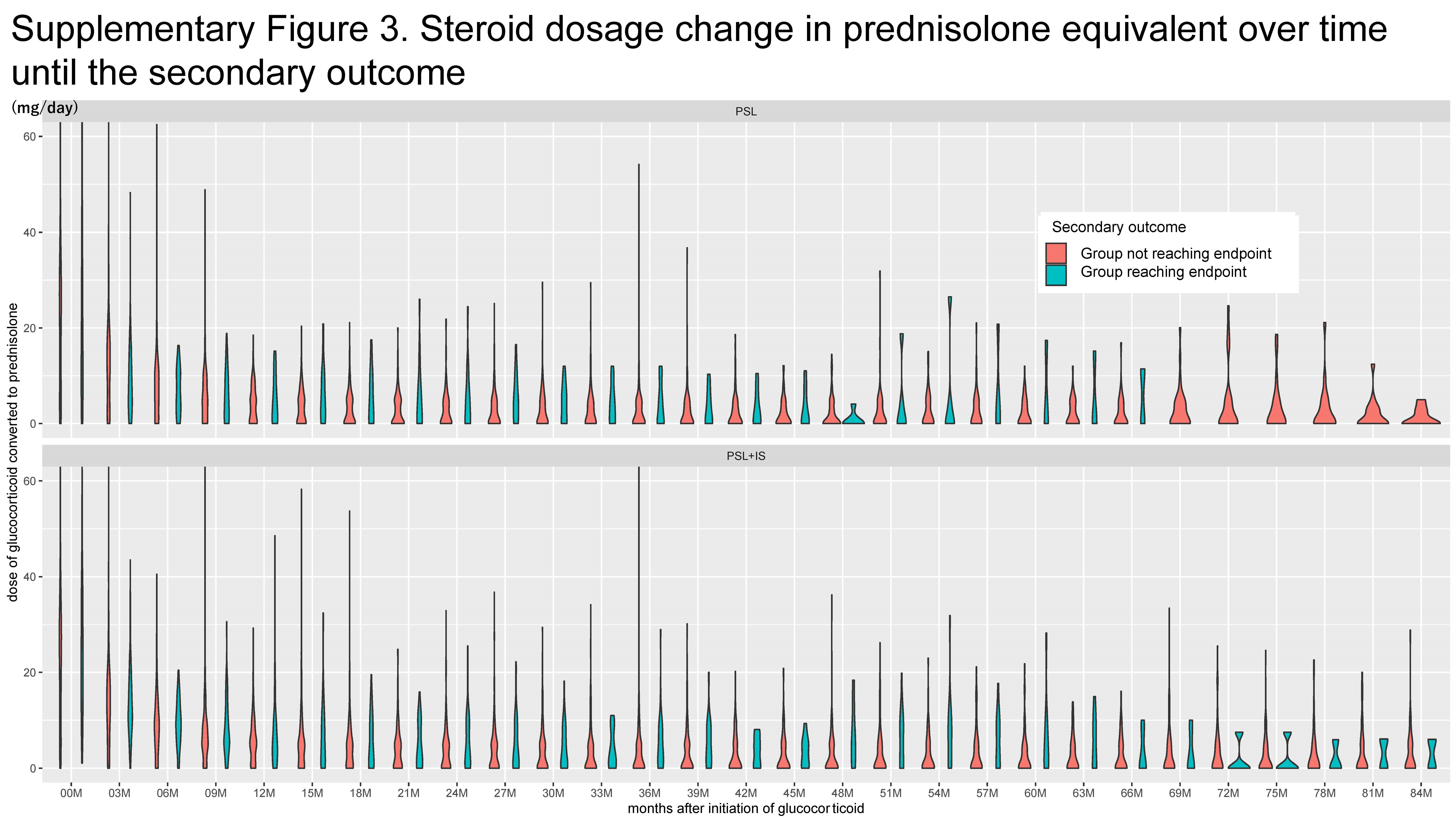


The blue area shows the group reaching endpoint. The red area shows the group not reaching endpoint. The upper panel shows the PSL group. The lower panel shows the PSL + IS group.

PSL, prednisolone; IS, immunosuppressive agent.
